# Community-Based Impact of Community-Based Antenatal Care Models on Prevention and Early Detection of Obstetric Complications: A Systematic Review

**DOI:** 10.5334/aogh.5356

**Published:** 2026-07-21

**Authors:** Oladiran Isdaiah Olagunju, Alangs Manasseh Stephen, Victoria Eleba Wokoma, Ibiang Okama Eko, Adegbite Khadijat Bolaji

**Affiliations:** 1 School of Community Health, Obafemi Awolowo University Teaching Hospitals Complex (OAUTHC), Ile-Ife, Osun State, Nigeria; 2 Department of Community Health, College of Community Health Sciences, Wesley University, Ondo State, Nigeria; 3 Department of Community Health, College of Health Technology, Calabar State, Nigeria

**Keywords:** community-based antenatal care, obstetric complications, early detection, maternal health, referral systems, LMICs, systematic review, community health workers

## Abstract

Obstetric complications remain a leading cause of preventable maternal and neonatal morbidity and mortality in low- and middle-income countries (LMICs). Persistent barriers to facility-based antenatal care (ANC), including geographic remoteness, transportation costs, socio-cultural norms, and fragmented health systems, have prompted the large-scale expansion of community-based ANC models designed to bring preventive and early-detection services directly to underserved populations. This systematic review evaluated the impact of community-based ANC models on the prevention and early detection of obstetric complications in LMICs, compared maternal and neonatal outcomes with conventional facility-based care, and identified critical implementation gaps.

A systematic review was conducted in accordance with PRISMA 2020 guidelines. Seven electronic databases, PubMed, CINAHL, Cochrane Library, WHO IRIS, Scopus, Embase, and Google Scholar, were searched for peer-reviewed studies published between 2000 and 2025. Studies were screened against predefined inclusion and exclusion criteria, and 40 studies that met these criteria were included. Data were synthesised both narratively and quantitatively.

The study concluded that 40 included studies (77.5%) reported significant improvements in preventive maternal health practices, including anaemia screening, blood pressure monitoring, and birth preparedness, attributable to community-based interventions. Early ANC initiation improved in 82.5% of studies, with referral compliance increasing by 18%–38% in community-supported models. Neonatal mortality reductions of 15%–24% were documented in participatory women’s group interventions. However, 65% of studies identified implementation challenges, including weak referral systems, inadequate community health worker (CHW) supervision, supply chain inconsistencies, and entrenched socio-cultural barriers.

Community-based ANC models greatly improve prevention and early detection of obstetric complications, especially in rural and resource-limited areas. Their effectiveness is maximised when they are organisationally integrated with primary healthcare systems, supported by strong referral connections, and tailored to local contexts. Expanding these models requires ongoing investment in the workforce, training, and coordination mechanisms for community facilities.

## Introduction

Maternal mortality remains one of the most distressing and largely preventable global health crises of the twenty-first century [1]. Despite decades of targeted interventions and the adoption of multiple international health frameworks, from the Millennium Development Goals to the Sustainable Development Goals (SDGs), the global maternal mortality ratio (MMR) remains unacceptably high, especially in low- and middle-income countries (LMICs). According to the World Health Organization [2], approximately 295,000 women died during or after pregnancy and childbirth in 2017 alone, with 94% of these deaths occurring in LMICs. Sub-Saharan Africa and South Asia together account for nearly 87% of global maternal deaths, highlighting deep structural inequities in health system access, quality, and responsiveness [2]. These statistics highlight an urgent need to rethink how antenatal care (ANC) is conceived, delivered, and assessed across diverse resource settings. Neonatal mortality is equally concerning. Each year, around 2.5 million newborns die within the first 28 days of life, most in LMICs, and a significant proportion of these deaths are linked to preventable obstetric complications during pregnancy and childbirth [3]. Preterm birth, birth asphyxia, neonatal sepsis, and low birth weight—conditions closely associated with poorly managed maternal health—constitute the majority of neonatal mortality worldwide [3]. The inextricable link between maternal and newborn survival means that enhancing the quality and reach of ANC is among the most impactful investments in global health. However, access to early, consistent, and high-quality ANC remains profoundly unequal, hindered by the same structural factors that undermine maternal health outcomes more broadly [4, 5]. The five principal direct causes of maternal mortality are haemorrhage, hypertensive disorders (including preeclampsia and eclampsia), sepsis, obstructed labour, and unsafe abortion. For clarity, this review distinguishes between obstetric complications (the direct clinical conditions that arise during pregnancy, labour, or the postpartum period, such as haemorrhage or sepsis), maternal morbidity (the short- or long-term health consequences that may result from such complications), and maternal mortality (death resulting from these complications). Collectively, these complications account for over 75% of all maternal deaths globally [1, 6]. What makes this figure particularly tragic is that most of these deaths are preventable through timely, evidence-based ANC and obstetric management. Haemorrhage, for example, can be significantly reduced through early detection of anaemia, iron supplementation, and birth preparedness planning, core functions of quality ANC [1, 6]. Similarly, hypertensive disorders during pregnancy can be identified and managed early via regular blood pressure monitoring and urine testing during antenatal visits [7].

The issue, however, is not merely a lack of knowledge about effective interventions. The real challenge is translating that knowledge into accessible, high-quality services that reach women at the right moment, ideally before complications become life-threatening emergencies. In many LMICs, the health system is not set up to ensure this timely access, especially for women in rural, peri-urban, or socially marginalised communities. The gap between what is clinically possible and what is epidemiologically achieved results directly from structural barriers that community-based models of care are specifically designed to address [8, 9]. Traditional facility-based ANC, while clinically effective in ideal conditions, faces well-documented constraints that limit its reach, uptake, and impact in resource-limited settings. Geographic distance is among the most persistent barriers: in many parts of sub-Saharan Africa and South Asia, health facilities are located several kilometres from rural communities, and the absence of reliable, affordable transportation effectively excludes large populations of pregnant women from accessing services [10, 11]. Economic barriers compound geographic ones: user fees, indirect costs such as transport and food, and the opportunity cost of attending multiple ANC visits during working hours are prohibitive for households in poverty.

Socio-cultural barriers represent another significant layer of constraint. In many contexts, women lack the autonomy to make independent decisions regarding healthcare seeking; male partner approval, extended family expectations, and traditional birthing practices all influence whether and when women initiate ANC [12, 13]. Health system issues, including staff shortages, poor quality of care, disrespectful treatment, long waiting times, and supply chain disruptions, further diminish women’s motivation to attend recommended ANC visits [14, 15]. The combined effect of these overlapping barriers means that many high-risk pregnancies remain undetected and unmanaged until complications become severe, making interventions more complicated and less likely to succeed. In response to these documented limitations of facility-based care, community-based ANC models have gained significance as a complementary, and sometimes transformative, approach to enhancing maternal and neonatal health outcomes. These models include a variety of service delivery strategies, such as community health worker (CHW) home visitation programmes, participatory women’s group sessions, mobile health (mHealth) interventions, and integrated community-facility outreach programmes. What unites these diverse approaches is their shared goal: to bring antenatal education, screening, and referral services closer to the households and communities where women live [9, 16].

CHWs, variously termed lady health workers, community midwives, village health volunteers, or health extension workers, depending on the national context, serve as the cornerstone of most community-based ANC programmes. Trained to perform basic maternal health screening, provide health education, and facilitate timely referral, CHWs act as a bridge between households and formal health facilities, significantly reducing both the physical and psychosocial distance between pregnant women and skilled care [17, 18]. Participatory women’s groups further augment CHW programmes by creating structured social learning environments in which women collectively build knowledge about danger signs, birth preparedness, and newborn care, thereby mobilising peer support networks that reinforce individual health-seeking behaviour [19]. Despite the documented benefits of these models, the evidence base remains fragmented. Significant variability exists across study designs, settings, and outcome measures, making it difficult to draw generalisable conclusions about the overall impact of community-based ANC on the prevention of obstetric complications and early detection. Furthermore, implementation challenges, including inadequate training, poor supervision, limited resources, and socio-cultural resistance, continue to undermine programme sustainability and scale-up in many contexts. This gap in systematic, synthesised evidence forms the primary motivation for the present review.

Obstetric complications continue to claim hundreds of thousands of maternal and neonatal lives each year, mostly in LMICs where conventional facility-based ANC is either inaccessible or grossly underutilised [2, 3]. While community-based ANC models have expanded as alternative delivery strategies, evidence of their effectiveness remains scattered across individual studies of varying methodological quality, design, and contextual relevance [8, 9]. Critical questions remain insufficiently addressed: Do community-based ANC models significantly reduce the incidence and severity of obstetric complications? Are they comparable to or more effective than facility-based care in rural and underserved settings? What are the main barriers to their implementation, and how do these barriers interact with health system capacity? The lack of a rigorous, comprehensive synthesis of this evidence creates a vital knowledge gap that hampers evidence-based policy-making, programme design, and resource allocation. Without such synthesis, policymakers and practitioners lack the consolidated guidance needed to expand, adapt, or strengthen community-based ANC interventions at scale. Therefore, this study aims to fill this gap by systematically reviewing and critically synthesising the global evidence on the impact of community-based ANC models on the prevention and early detection of obstetric complications. It seeks to evaluate systematically the effect of community-based ANC models on preventing obstetric complications, including preeclampsia, antepartum and postpartum haemorrhage, and sepsis; assess the role of community-based ANC in early identification and referral of high-risk pregnancies; compare maternal and neonatal outcomes between community-based ANC models and conventional facility-based care; and identify implementation gaps, contextual barriers, and facilitators influencing the effectiveness and sustainability of these interventions.

## Review

The relationship between community-level health interventions and maternal mortality outcomes has been extensively studied in LMICs over the past two decades, with accumulating evidence demonstrating that well-designed, contextually appropriate programmes can significantly reduce both maternal and neonatal deaths. A landmark systematic review conducted in sub-Saharan Africa found that community-based interventions, including CHW home visitation, community education groups, and integrated referral systems, were associated with statistically significant reductions in maternal mortality, with the most pronounced effects observed in rural and geographically isolated settings [20]. These findings align with earlier meta-analytic evidence from South Asia, where participatory women’s group programmes reduced maternal mortality by 37% in high-coverage rural populations [19]. A more recent systematic review focused on Lagos and Ogun States in Southwest Nigeria documented that CHWs trained in maternal danger-sign recognition and emergency referral contributed to measurable declines in MMRs in study communities [21]. Importantly, this review highlighted that effectiveness was contingent on structural integration with formal health services. This finding has been consistently replicated across geographic contexts, including studies from South Asia, sub-Saharan Africa, Southeast Asia, and Latin America (see Results for the full geographic distribution of included studies). Similarly, Aidoo [22] examined community-based healthcare interventions among minority and underserved populations, concluding that community health educators and peer support networks improved both maternal and infant health outcomes through sustained behavioural change and increased health service utilisation. These findings collectively suggest that the mechanism of impact operates less through the direct clinical activities of CHWs and more through the systemic change in health-seeking behaviour they facilitate.

### Antenatal care adherence and timing of ANC initiation

One of the most consistent findings in the literature is that community-based interventions significantly improve ANC adherence, measured by both the timing of the first ANC visit and the total number of visits completed [8, 9]. Late ANC initiation, defined as first booking in the second or third trimester, is a major predictor of undetected obstetric complications, as many high-risk conditions, including hypertensive disorders, anaemia, and foetal growth restriction, can only be identified and managed if screening begins in the first trimester. A study by Chilanga and Hazemba [23] examining late ANC booking at a rural health centre in Zambia found that socio-cultural factors, lack of husband support, inadequate community awareness, and long distances to health facilities were the primary drivers of delayed first ANC visits, precisely the barriers that community-based models are designed to mitigate.

In Kenya, Monda [24] evaluated a community-based intervention designed to improve adherence to ANC follow-up contacts among pregnant women in Nyamira County and found that CHW-led education and reminder visits significantly increased the proportion of women completing the WHO-recommended ANC schedule. These results are consistent with evidence from Bangladesh and Nepal, where home visitation programmes achieved 20%–45% improvements in first-trimester ANC booking rates [18, 19]. The implications for obstetric complication detection are direct: earlier and more frequent ANC contacts create more opportunities for screening, monitoring, and timely referral, all of which are foundational to preventing complications from reaching critical severity.

Stillbirth, defined as foetal death at 28 or more weeks of gestation, represents an under-acknowledged dimension of the obstetric complication burden in LMICs, with nearly 98% of the estimated 1.9 million annual stillbirths occurring in LMICs. A recent systematic review and meta-analysis by Gwacham-Anisiobi et al. [25] examined the effects of community-based interventions on stillbirths across sub-Saharan Africa, finding that integrated community-facility programmes incorporating antenatal home visits, birth preparedness education, and CHW-facilitated referral were associated with 15–28% reductions in stillbirth rates in intervention communities. The authors identified skilled birth attendance as the most critical mediating pathway between community-based antenatal programmes and stillbirth prevention.

These findings are particularly significant in the context of Ethiopia, where national data show that the place of maternal death and delivery is closely linked to ANC attendance and proximity to skilled care facilities [26]. Tesfay et al. [26] conducted a generalised structural equation modelling analysis of maternal deaths in Ethiopia. They found that women who died outside of health facilities had significantly lower rates of ANC attendance, underscoring the protective role of ANC in linking women to emergency obstetric services. Community-based models, by increasing ANC attendance and facilitating earlier contact with health systems, may therefore reduce out-of-facility maternal and perinatal deaths by ensuring that high-risk women are known to the health system before delivery.

### mHealth and technological innovations in community ANC

The integration of mHealth technologies into community-based ANC represents a rapidly evolving frontier in maternal health service delivery. A systematic review and meta-analysis by Rahman et al. [27] evaluated the effects of mHealth interventions, including SMS reminders, voice calls, mobile apps, and telemedicine consultations, on attendance at ANC visits and skilled delivery care in LMICs. The review found that mHealth interventions were associated with a 27% increase in the odds of completing 4 or more ANC visits and a 22% increase in the odds of attending a skilled birth attendant. These effects were particularly pronounced in contexts where digital connectivity had been integrated with CHW programmes, suggesting a complementary synergy between human and technological components of community ANC systems [27].

The potential for mHealth to scale community-based ANC without proportional increases in human resource costs is especially relevant for LMICs with critical shortages of trained healthcare workers. However, the same review identified persistent digital divide concerns—particularly for women with limited literacy, older mobile technology, or unreliable network connectivity—that must be addressed in mHealth programme design to avoid widening existing health inequities. Marks and Castro [28] further underscore the need for context-specific community-based approaches in their narrative review on maternity waiting homes in Latin America, demonstrating that even geographically focused solutions such as maternity waiting homes, which serve as community-based transit points linking rural women to facility-based delivery, can significantly reduce delays in accessing skilled care and reduce maternal morbidity in high-altitude and remote settings.

### Gaps

While the foregoing literature demonstrates a robust and growing body of evidence supporting the potential of community-based ANC models to improve maternal and neonatal health outcomes, several critical gaps persist. First, the majority of existing systematic reviews focus on specific intervention types, such as CHW programmes or participatory women’s groups, in isolation, rather than synthesising evidence across the full spectrum of community-based ANC modalities. Second, comparative analyses of community-based versus facility-based ANC, particularly in diverse contextual and health system conditions, remain limited in scope and methodological rigour. Third, implementation science perspectives, including the factors that enable or constrain the sustainability and scale-up of community-based ANC, are insufficiently represented in the existing literature. This systematic review addresses these gaps by providing a comprehensive, multi-intervention synthesis of evidence on the effectiveness of community-based ANC, comparative outcomes, and implementation determinants in LMICs.

## Method

### Study design

This study employed a systematic review design and was conducted in accordance with the PRISMA 2020 (Preferred Reporting Items for Systematic Reviews and Meta-Analyses) guidelines [29]. The systematic review method was selected for its ability to rigorously synthesise evidence from multiple studies, minimise selection bias through transparent, reproducible search and inclusion processes, and provide a comprehensive understanding of the impact of community-based ANC models across diverse settings and populations. A narrative and quantitative synthesis approach was adopted to accommodate the heterogeneity of study designs and outcome measures in the included literature.

### Search strategy and databases

A comprehensive search of electronic databases was conducted to identify relevant peer-reviewed studies. The databases searched included: PubMed/MEDLINE, CINAHL (Cumulative Index to Nursing and Allied Health Literature), the Cochrane Library, the World Health Organization’s Institutional Repository for Information Sharing (WHO IRIS), Scopus, Embase, and Google Scholar. Search terms were developed using a PICO (Population, Intervention, Comparison, Outcome) framework, combining Medical Subject Headings (MeSH) and free-text terms. Key search terms included: ‘community-based antenatal care’, ‘antenatal care models’, ‘obstetric complications’, ‘maternal health’, ‘community health workers’, ‘home visitation’, ‘participatory women’s groups’, ‘low- and middle-income countries’, ‘maternal mortality’, ‘neonatal mortality’, ‘early detection’, and ‘referral’. Boolean operators (AND, OR) were applied to combine terms across domains. Reference lists of included studies and relevant systematic reviews were also hand-searched to identify any additional eligible studies not captured through database searches.

### Inclusion and exclusion criteria

Studies were included if they: (1) evaluated any community-based ANC intervention in a LMIC setting; (2) reported outcomes related to obstetric complication prevention, early detection of high-risk pregnancies, maternal morbidity or mortality, neonatal morbidity or mortality, or referral compliance; (3) employed randomised controlled trial, quasi-experimental, cohort, case-control, cross-sectional, or systematic review designs; and (4) were published in peer-reviewed journals in the English language between January 2000 and December 2025. Studies were excluded if they focused exclusively on facility-based ANC without a community component, were conducted in high-income countries without explicit comparison to LMIC contexts, were grey literature without peer review, or lacked sufficient outcome data for quality appraisal.

### Study selection and data extraction

Following the database search, duplicate records were removed using citation management software. Two independent reviewers screened all retrieved titles and abstracts against the eligibility criteria, with disagreements resolved through consensus discussion and, where necessary, arbitration by a third reviewer. Full-text articles were retrieved for all studies that passed abstract screening, and each was subjected to full-text eligibility assessment. A standardised data extraction form was developed and piloted across five studies before being applied to the full set of included studies. Data extracted for each study included: study location, design, sample size, type of community-based ANC intervention, comparison group, primary and secondary outcomes measured, and key findings.

### Quality assessment

The methodological quality of included studies was assessed using validated appraisal tools appropriate to each study design. Randomised controlled trials were assessed using the Cochrane Risk of Bias Tool 2.0; quasi-experimental and observational studies were appraised using the Newcastle-Ottawa Scale; and systematic reviews were evaluated using the AMSTAR-2 (A Measurement Tool to Assess Systematic Reviews) checklist. Studies rated as critically low quality on any appraisal tool were excluded from the quantitative synthesis, though they were retained in the narrative synthesis to provide contextual evidence.

### Data synthesis

Given the heterogeneity in intervention types, study designs, and outcome definitions across included studies, a dual synthesis strategy was employed. A narrative synthesis was conducted for all included studies, organised thematically around the four specific objectives of the review. For studies reporting comparable quantitative outcomes, particularly neonatal mortality rates, ANC attendance rates, and referral compliance, a quantitative synthesis was performed using pooled proportions and narrative summaries of effect sizes. Where sufficient homogeneity was present in study designs and outcomes, random-effects meta-analytic models were applied in Stata 17.0. Statistical heterogeneity was assessed using the I² statistic, with values above 75% indicating substantial heterogeneity; in such cases, results were interpreted with caution, and subgroup analyses were conducted to explore sources of variation.

### Ethical considerations

As a secondary review of published literature, this study did not involve primary data collection and was therefore exempt from institutional ethical review requirements. All included studies underwent ethical scrutiny at the time of their original publication. Data were handled in accordance with principles of academic integrity, with all sources fully cited and all authors’ intellectual contributions acknowledged. The review protocol was prospectively registered on PROSPERO (International Prospective Register of Systematic Reviews) prior to commencement of the formal review process.

## Results

### Study selection and characteristics

The systematic database search identified a total of 2,847 records across the seven databases. After removing duplicates (*n* = 612), 2,235 unique records were screened at the title and abstract levels. Of these, 1,894 were excluded as clearly irrelevant to the review objectives. A total of 341 full-text articles were retrieved and assessed for eligibility, of which 301 were excluded for the following reasons: exclusive focus on facility-based ANC without community component (*n* = 89), conducted in high-income countries (*n* = 67), insufficient or inadequate outcome data (*n* = 73), grey literature without peer review (*n* = 42), and non-English language (*n* = 30). Forty studies met all inclusion criteria and were included in the final synthesis. The PRISMA flow diagram documents the complete selection process.

The 40 included studies comprised 14 randomised controlled trials (35%), 11 quasi-experimental studies (27.5%), 8 observational cohort or cross-sectional studies (20%), and 7 systematic reviews or meta-analyses (17.5%). Geographically, the studies were distributed across South Asia (*n* = 17, 42.5%), sub-Saharan Africa (*n* = 16, 40%), Southeast Asia (*n* = 4, 10%), and Latin America (*n* = 3, 7.5%). The study period ranged from 2003 to 2024. Sample sizes varied widely, from 245 participants in smaller quasi-experimental studies to 1.2 million participants in large multi-country meta-analyses. The most common community-based ANC interventions studied were CHW home visitation programmes (*n* = 22), participatory women’s groups (*n* = 18), integrated community-facility outreach (*n* = 14), and mHealth-supported community ANC (*n* = 9). Some studies evaluated combinations of these intervention types.

### Impact on prevention of obstetric complications

Of the 40 included studies, 31 (77.5%) reported statistically significant improvements in one or more preventive maternal health practices attributable to community-based ANC interventions. The most consistently improved preventive outcomes were: iron-folic acid supplementation uptake (reported in 19 studies, with improvements ranging 18%–44%), blood pressure monitoring compliance (17 studies; 22%–38% improvement), anaemia screening coverage (15 studies; 25%–45% improvement), and birth preparedness behaviours (20 studies; 20%–55% improvement). These findings indicate that community-based models effectively address several of the most modifiable risk factors for major obstetric complications.

In relation to specific obstetric complications, evidence was strongest for the prevention of hypertensive disorders and anaemia-related complications. Studies incorporating regular CHW-led blood pressure monitoring and referral showed a 24%–32% reduction in the incidence of severe preeclampsia reaching clinical facilities in a critical state, primarily through early detection and timely referral before conditions escalated [9, 17]. Anaemia prevention was similarly improved: a cluster-randomised trial in Bangladesh found that CHW-supported distribution of iron-folic acid and dietary counselling reduced the prevalence of moderate-to-severe anaemia by 31% in intervention communities compared with controls [18].

Evidence for the prevention of sepsis and infection-related complications was less consistent, primarily because many community-based programmes lack the clinical capacity to detect and treat infections. However, studies incorporating hygiene education, clean delivery kit distribution, and community referral protocols showed modest improvements in infection-related neonatal outcomes, suggesting that community-based programmes can contribute to infection prevention through behavioural and environmental pathways, even in the absence of clinical treatment capacity.

### Early detection and referral of high-risk pregnancies

Across the included studies, community-based ANC consistently demonstrated a significant capacity to improve early detection of high-risk pregnancies and to facilitate timely referral to skilled care. Improvements in early ANC initiation were documented in 33 of 40 studies (82.5%), with the timing of first ANC visit shifting from the second or third trimester to the first trimester in 20%–45% of eligible women in intervention communities. This represents a clinically meaningful shift, as first-trimester ANC attendance creates opportunities for early screening for hypertensive disorders, foetal growth abnormalities, multiple pregnancies, and pre-existing medical conditions that substantially elevate obstetric risk. Knowledge of obstetric danger signs, including heavy vaginal bleeding, severe headache, blurred vision, convulsions, high fever, and absent or reduced foetal movements, improved markedly in communities with active CHW education programmes, with correct identification of at least three danger signs increasing by 35%–60% in intervention versus control communities [30, 31]. This improvement in knowledge was associated with a 22%–38% increase in referral compliance, meaning that women identified as high risk were more likely to follow through with their referral to a higher-level facility. Critically, however, referral effectiveness, the proportion of referred women who actually received appropriate care at the referral facility, was substantially lower in settings with weak transport infrastructure, facility overcrowding, or insufficient emergency obstetric care capacity [15, 32].

### Comparative maternal and neonatal outcomes

Of the 40 included studies, 16 provided direct comparisons between community-based ANC models and conventional facility-based care, enabling an assessment of their relative effectiveness. The evidence from these comparative studies was nuanced, with community-based models demonstrating superiority in rural and underserved contexts but near-equivalence in urban or better-resourced settings. In rural contexts, community-based ANC was associated with 15%–24% reductions in neonatal mortality compared to standard facility-based care controls, consistent with the meta-analytic findings of Prost et al. [19] and Gwacham-Anisiobi et al. [25]. MMRs were also lower in intervention communities, though the effect size varied by intervention intensity and health system context. Adherence to the WHO-recommended ANC schedule (four or more visits, or the updated standard of eight or more contacts) improved by 22%–35% in community-supported models compared to facility-only controls [2]. In rural Nepal, a multi-arm RCT comparing women’s group ANC with standard government facility ANC found that the women’s group model was associated not only with improved neonatal survival but also with greater maternal empowerment, increased utilisation of skilled birth attendants, and reduced reporting of disrespectful or abusive maternity care. In summary, this intervention was found to be effective in a low-resource, rural LMIC setting where facility-based alternatives were limited.

In urban and peri-urban settings, where geographic and transportation barriers are less severe and access to facilities is relatively better, the comparative advantage of community-based ANC was attenuated. Studies in urban Nigeria and India found that while community-based programmes improved health literacy and care-seeking behaviour, maternal and neonatal outcomes did not differ significantly from those observed under standard facility-based ANC [10, 33].

### Implementation gaps and challenges

Among the included studies, 65% (*n* = 26) identified significant implementation challenges affecting the effectiveness, sustainability, or equitable reach of community-based ANC programmes. The most frequently cited barriers were: inadequate CHW training and competency development (reported in 18 studies), insufficient or irregular supervision and performance support (15 studies), inconsistent supply chains for essential maternal health commodities (14 studies), and weak or non-functional referral systems (13 studies). Socio-cultural barriers, including male partner dominance in healthcare decision-making, religious and traditional birth practices, and stigma around certain pregnancy-related conditions, were identified as significant constraints in 17 studies, consistent with findings from the broader literature on maternal healthcare utilisation in LMICs [34, 35]. Financial sustainability emerged as a recurrent concern, with many community-based programmes demonstrating excellent short-term outcomes during funded project periods but experiencing rapid deterioration in service quality and coverage after external funding was withdrawn. Few programmes had developed sustainable domestic financing mechanisms or achieved full integration into national health budgets, raising serious questions about long-term impact and scalability. Governance challenges, including unclear role delineation between CHWs and other health workers, limited community ownership of programmes, and insufficient coordination between community and facility levels, further complicated implementation in several country contexts.

Facilitators of effective implementation included: strong community ownership and leadership engagement; clearly defined CHW roles with adequate remuneration and recognition; robust supervisory structures with regular supportive visits; functional referral linkages backed by community emergency transport schemes; and political commitment at national and district levels. Programmes that invested in formative community engagement and co-design processes achieved greater local acceptance and higher CHW retention rates, suggesting that participatory programme development is not merely an ethical preference but a practical determinant of programme effectiveness.

## Discussion

### Community-Based ANC and prevention of obstetric complications

The findings of this systematic review provide compelling evidence that community-based ANC models are effective in improving preventive maternal health behaviours and reducing the risk of specific obstetric complications, particularly in rural and resource-limited settings. The 77.5% rate of studies reporting significant improvements in at least one preventive outcome, blood pressure monitoring, anaemia screening, iron supplementation, and birth preparedness, confirms that community-based programmes, when well-designed and adequately resourced, can meaningfully shift the population-level distribution of key obstetric risk factors. This finding is consistent with the broader maternal health literature, which has repeatedly demonstrated that preventive interventions delivered at the community level can reduce the incidence of life-threatening complications without requiring proportional expansions in facility-based infrastructure [7, 9]. The evidence on anaemia prevention is particularly striking in its implications for haemorrhage-related maternal mortality. Given that iron-deficiency anaemia affects an estimated 38% of pregnant women globally, with prevalence exceeding 50% in some sub-Saharan African and South Asian contexts, the 31% reduction in moderate-to-severe anaemia documented in CHW-supported programmes represents a potentially massive impact on maternal mortality risk at the population scale. CHWs’ capacity to deliver nutritional counselling, distribute iron-folic acid supplements, and monitor compliance during home visits addresses a critical supply-side gap that facility-based ANC cannot efficiently bridge for women who are unable or unwilling to attend regular clinic appointments. Findings from Oluwole et al. [21] in Southwest Nigeria provide important context for these global patterns, demonstrating that even within a single country, the impact of community-based interventions varies significantly by state-level health system capacity, the quality of community engagement, and the adequacy of CHW training. This variability reinforces the importance of contextual adaptation and quality assurance in community-based programme design, and cautions against uncritical replication of models that have succeeded in one context without careful assessment of transferability.

### Early detection, referral systems, and the three delays

The consistent improvement in early ANC initiation and recognition of danger signs documented in 82.5% of included studies directly addresses the first and second delays in the Three Delays Model [36]. By equipping women with knowledge of pregnancy danger signs and empowering them to seek care without waiting for household or community permission, community-based ANC programmes interrupt the social and informational mechanisms that drive delayed care-seeking. The 35%–60% improvement in correct identification of danger signs is particularly noteworthy, as it suggests that community-based education is substantially more effective at translating health information into protective knowledge than one-off facility-based consultations, which are often constrained by time pressure and communication barriers. The 22%–38% improvement in referral compliance represents a meaningful advance in reducing the second delay—the time between recognising a problem and reaching skilled care. However, the substantial gap between referral compliance and referral completion (the actual receipt of appropriate care at a referral facility) reveals the persistent challenge of the third delay, which is driven by factors internal to the health system and therefore outside the influence of community-based programmes alone. Programmes that achieved the highest referral completion rates did so by explicitly addressing third-delay factors: establishing formalised communication channels between CHWs and facility staff, creating community emergency funds for transport costs, and ensuring that referred women were triaged promptly upon arrival rather than placed at the back of standard queues. These hybrid community-facility strategies represent a model of integrated care that is more effective than either community-based or facility-based approaches in isolation [37, 38].

The evidence from Chilanga and Hazemba [23] on late ANC booking in Zambia illustrates the complex, multi-layered nature of the barriers to early ANC, even in settings where community-based programmes have been implemented. Their finding that socio-cultural factors and male partner influence remain dominant determinants of ANC timing, even when geographic and financial barriers have been partially addressed, points to the need for community-based programmes to invest more substantially in gender-transformative approaches that engage men and community leaders as active advocates for maternal health, rather than treating male influence as an immutable barrier.

### Comparative effectiveness: Community-based vs facility-based ANC

The comparative analysis in this review reveals a consistent pattern: community-based ANC models demonstrate their greatest comparative advantage in rural, geographically isolated, and socio-economically disadvantaged settings where facility-based care is genuinely inaccessible or unutilised. In these contexts, the alternative to community-based ANC is frequently no ANC at all or care in a poorly resourced facility, meaning that the relevant comparison for policy purposes is not community-based versus facility-based, but community-based versus no care. The 15%–24% reductions in neonatal mortality achieved by participatory women’s group programmes in rural Nepal, India, Bangladesh, and Malawi [19, 25] are therefore best interpreted as evidence of community-based models filling a critical service gap, rather than as evidence that they are inherently superior to well-resourced facility-based care. In urban and better-resourced settings, comparative evidence suggests that community-based ANC is best positioned as a demand-side complement to facility-based supply-side investments. By improving maternal health literacy, addressing psychosocial barriers to care-seeking, and facilitating linkages between households and facilities, community-based programmes can substantially increase the effective utilisation of existing facility-based ANC services. This role is valuable precisely because large investments in maternal health facility infrastructure often fail to translate into commensurate improvements in population-level outcomes without corresponding investments in demand generation and patient navigation. It is important to note, however, that the magnitude and consistency of these effects are not uniform across health systems. Countries with decentralised governance, established CHW cadres, and stronger domestic financing for primary care, such as Ethiopia and Nepal, were better able to integrate community-based ANC into existing service structures formally. In contrast, countries with more fragmented, donor-dependent, or facility-centric health systems showed greater variability in outcomes and sustainability. Political will and the existence of a supportive policy environment, including national CHW cadres, standardised supervision structures, and dedicated budget lines, therefore, appear to moderate the effectiveness of community-based ANC at least as much as the intrinsic design of the intervention itself, underscoring that transplanting a successful model into a different health system context is unlikely to succeed without equivalent investment in the surrounding system [39].

### Implementation challenges

The finding that 65% of included studies identified significant implementation challenges highlights a critical gap between the proven potential of community-based ANC and its actual delivery in practice. CHW training quality, supervision intensity, and supply chain reliability emerged as the three most modifiable implementation determinants in this review, and their consistent citation across geographically and culturally diverse settings suggests that they represent universal, rather than context-specific, programme quality requirements. The evidence from Monda [24] in Kenya and Orjingene and Morgan [20] in sub-Saharan Africa collectively points to a common pattern: programmes that invest in regular, supportive supervision and ongoing competency-based training sustain their impact over time, while those that treat CHW training as a one-time event and rely on informal, unsupported community implementation experience rapid quality deterioration.

Financial sustainability represents perhaps the most challenging implementation barrier identified in this review. The dependence of most community-based ANC programmes on short-term donor funding creates an inherent structural vulnerability that undermines both long-term impact and national health system strengthening. Promising approaches to sustainability include: integration of CHW remuneration into national health workforce frameworks, incorporation of community-based ANC performance metrics into national health information systems, and development of community financing mechanisms, such as community-based health insurance schemes, that can sustain programme operations beyond project periods. The evidence base for these sustainability strategies remains thin, representing an important area for future research and policy development.

### Strengths and limitations

This systematic review has several notable strengths. It employed a comprehensive, multi-database search strategy with prospective protocol registration; applied validated quality appraisal tools appropriate to different study designs; and synthesised evidence across a broad range of intervention types, geographic settings, and outcome domains. The inclusion of both quantitative and narrative syntheses enabled a nuanced analysis that captured the magnitude and contextual variability of the impact of community-based ANC. Limitations include the potential for publication bias, as studies with null or negative findings may be less likely to be published; the heterogeneity in intervention definitions and outcome measurements that complicated quantitative pooling; and the predominance of studies from a limited number of countries (particularly Nepal, Bangladesh, India, and sub-Saharan Africa), which may limit the generalisability of findings to other LMIC contexts with different health system structures and epidemiological profiles.

## Conclusion

This systematic review has provided a comprehensive synthesis of the evidence on the impact of community-based ANC models on the prevention and early detection of obstetric complications in LMICs. The findings demonstrate that community-based ANC models, encompassing CHW home visitation, participatory women’s groups, mHealth interventions, and integrated community-facility outreach, significantly improve preventive maternal health behaviours, increase early initiation of ANC, enhance recognition of danger signs, and reduce neonatal mortality, particularly in rural and resource-limited settings. These benefits operate through reductions in the first and second delays within the Three Delays framework. They are most pronounced in contexts where community-based programmes are structurally integrated with responsive, capable health facility systems. The evidence also reveals persistent implementation challenges, including inadequate training, weak supervision, unsustainable financing, and socio-cultural barriers, that limit the full realisation of the potential of community-based ANC. Closing the gap between demonstrated effectiveness under research conditions and consistent quality at scale requires sustained investment in health system strengthening, human resource development, community engagement, and governance. Community-based ANC should be unequivocally recognised not as a peripheral or temporary add-on to formal health systems, but as a structurally essential component of comprehensive maternal health strategies in LMICs.

## Recommendations

### Government and policymakers

Government and policymakers should formally integrate community-based ANC into national maternal health policies, ensure dedicated funding in domestic health budgets, and establish clear quality standards for CHW training, supervision, and performance monitoring. In addition, there is a need to strengthen the referral system infrastructure by improving communication between communities and health facilities, implementing emergency transport schemes, and enhancing emergency obstetric care capacity, so that early detection at the community level is effectively supported by timely facility-based care. Furthermore, policymakers should develop and evaluate context-specific sustainable financing models, such as integrating CHWs into the formal health workforce and promoting community health insurance schemes, to reduce reliance on short-term external donor funding.

### Healthcare practitioners

Healthcare practitioners should adopt gender-transformative approaches that actively involve male partners and community leaders as advocates for ANC, thereby addressing socio-cultural barriers that limit women’s autonomy in seeking care. They should also prioritise integrating mHealth tools into community ANC programmes, ensuring that these digital interventions are designed collaboratively with communities to address literacy levels and poor network connectivity, which could otherwise exacerbate health inequities.

### Future research

The research should focus on conducting comparative effectiveness studies using standardised definitions of interventions and consistent outcome measures across diverse LMIC settings to allow for stronger cross-study comparisons. Additionally, there is a need to invest in implementation science research to identify the essential support conditions required for community-based ANC programmes to achieve and sustain optimal effectiveness when scaled up.
